# The impact of media composition on production of flavonoids in agitated shoot cultures of the three *Hypericum perforatum* L. cultivars ‘Elixir,’ ‘Helos,’ and ‘Topas’

**DOI:** 10.1007/s11627-018-9900-7

**Published:** 2018-04-09

**Authors:** Inga Kwiecień, Julia Smolin, Ludger Beerhues, Halina Ekiert

**Affiliations:** 1 Department of Pharmaceutical Botany, Jagiellonian University, Medical College, Medyczna street 9, 30-688 Kraków, Poland; 2 Institute of Pharmaceutical Biology, Technische Universität Braunschweig, Mendelssohnstrasse 1, 38106 Braunschweig, Germany

**Keywords:** Flavonoid aglycones, Flavonoid glycosides, *In vitro* cultures, Plant growth regulators, Basal media composition

## Abstract

The aims of the study were to evaluate the effect of media composition on the growth potential and morphology of the *in vitro* cultured biomass of three cultivars of *Hypericum perforatum*, and on the production of flavonoids. Agitated shoot cultures were maintained in parallel on Linsmaier and Skoog (LS) and Murashige and Skoog (MS) media supplemented with 0.1–3.0 mg L^−1^ of α-naphthaleneacetic acid and 6-benzylaminopurine. Methanolic extracts from the biomass collected after 3-wk growth cycles were analyzed quantitatively, for 21 flavonoids using high performance liquid chromatography. Three aglycones (kaempferol, luteolin, and quercetin) and three glycosides of quercetin (hyperoside, quercitrin, and rutoside) were detected in all of the extracts. The total amounts of the estimated compounds increased from 1.18- to 21.66-fold on LS media variants and from 1.52- to 17.34-fold on MS media variants. The main metabolite was quercetin (max. 210.55 mg 100 g^−1^ dry weight [DW]). The maximum total amounts of all compounds in the biomass of ‘Elixir,’ ‘Helos,’ and ‘Topas’ were 328.53, 255.70, and 166.58 mg 100 g^−1^ DW, respectively. The shoots of all cultivars cultivated on the LS and MS media containing low levels of plant growth regulators (0.1 mg L^−1^) accumulated high amounts of flavonoids. The highest amounts were accumulated in shoots of cultivar ‘Elixir’ grown on MS medium. This is the first comparison of flavonoid production in three cultivars of *H. perforatum* (‘Elixir,’ ‘Helos,’ and ‘Topas’) cultured *in vitro*, and the first report of flavonoid production in cultivars ‘Elixir’ and ‘Helos.’

**Electronic supplementary material:**

The online version of this article (10.1007/s11627-018-9900-7) contains supplementary material, which is available to authorized users.

## Introduction

Flavonoids, as polyphenolic plant metabolites, have been an object of special interest in recent years due to their antioxidant properties. These compounds have a number of additional biological properties, which are extremely important in therapy and cosmetology, including sealing blood vessels, preventing the aggregation of platelets, and preventing inflammation. Due to the antioxidant properties of flavonoids, these metabolites prevent cardiovascular disorders, oncological processes, and possess anti-aging activities (Rice-Evans et al. 1996; Kumar and Pandey 2013).

A rich source of flavonoids, among plant species, is *Hypericum perforatum* L. (St. John’s Wort). It is a very well-known medicinal plant species, which is popular in traditional and modern phytomedicine (European Pharmacopoeia 9.0 2016). Apart from flavonoids, the herb contains hypericins, tannins, phenolic acids, hyperforins, xanthones, and terpenoids. Flavonoids are represented by aglycones, such as kaempferol, quercetin, and luteolin and their glycosides, along with some dimeric flavonoids (Jürgenliemk and Nahrstedt 2002; Barnes et al. 2007; Cirak et al. 2007). Hyperoside, the primary *H. perforatum* flavonoid compound in the plant extracts, ranges from 1.7 mg 100 g^-1^ to 4.0 g 100 g^−1^ dry weight (DW) (Jürgenliemk and Nahrstedt 2002; Cirak et al. 2007; Silva et al. 2008).

The biosynthesis and the accumulation of metabolites, including flavonoids, in soil-grown plants, are affected by growing conditions, such as geographical origin, habitat conditions, and population diversity of the plants growing in the wild (Fari et al. 2006; Bruni and Sacchetti 2009). The use of biotechnological methods makes it possible theoretically and often practically, to omit all abovementioned problems connected with differences in chemical composition, and therapeutical value of plant raw material. *In vitro* cultures of medicinal plant species provide the possibility to control and stimulate the biosynthesis, and consequently the accumulation of secondary metabolites, which are important in therapy. *In vitro* biomass could be a better source of metabolites than soil-grown plants. The *in vitro* cultures of many medicinal plants could accumulate high levels of different groups of plant metabolites with antioxidant activity, including flavonoids (Matkowski 2008).

Several biotechnological studies of *H. perforatum* and other *Hypericum* species focused on biosynthesis and accumulation of anthraquinone and phloroglucinol derivatives, or xanthones (Pasqua et al. 2003; Gadzovska et al. 2005; Beerhues 2011; Tusevski et al. 2013a, 2015, 2016; Simic et al. 2015). Biosynthesis and accumulation of flavonoids in *H. perforatum in vitro* systems were also studied. Other research studies revealed a number of flavonoid aglycones and glycosides, especially in shoot culture systems (Dias et al. 1999; Pasqua et al. 2003; Bertoli et al. 2008). Some publications reported on *H. perforatum in vitro* cultures producing high levels of flavonoids, especially in plant biomass with a high degree of organogenesis (shoot cultures), and/or after genetic transformation (Tusevski et al. 2013b, 2014). It was also reported that in *H. perforatum* cultivar ‘Topas’ cultures characterized by high metabolite production, there was an absence of the leading *H. perforatum* metabolites rutoside and hyperoside, especially in undifferentiated *in vitro* cultures (Pasqua et al. 2003). Apart from this cultivar, other well-known *H. perforatum* cultivars with valuable properties were the object of the present study, including ‘Elixir,’ a producer of high levels of biologically active secondary metabolites, and ‘Helos,’ a cultivar highly resistant to fungal pathogens.

Among the factors affecting the biosynthesis and accumulation of secondary metabolites in *in vitro* cultures, are the basal medium composition and the composition and concentrations of plant growth regulators (PGRs) (Ramawat and Mathur 2007; Danova et al. 2012). The type of culture is also important. Microshoot cultures should be tested because the cells in such a highly differentiating *in vitro* system can possess high growth potential and can produce high levels of biologically active metabolites.

Earlier studies of *in vitro* cultures of cultivars ‘Elixir,’ ‘Helos,’ and ‘Topas’ focused on the analysis of non-hallucinogenic indole compounds and free phenolic acids in agar and agitated *in vitro* cultures, respectively. High but different biosynthetic potential of cells of the three cultivars were documented (Muszyńska et al. 2014; Kwiecień et al. 2015). These interesting results encouraged the investigation into the biosynthetic potential for flavonoid production. To assess the biogenetic potential of cells, the cultivars were analyzed for hypericin content. The aim of the present research was to evaluate the effect of Linsmaier and Skoog (LS; Linsmaier and Skoog 1965) and Murashige and Skoog (MS; Murashige and Skoog 1962), basal media, and α-naphthaleneacetic acid (NAA) and 6-benzylaminopurine (BAP) on the production of bioactive flavonoids, by cells from *in vitro* cultures in an agitated *in vitro* system of *H. perforatum* cultivars ‘Elixir,’ ‘Helos,’ and ‘Topas’ using a high-performance liquid chromatography (HPLC) technique. This is the first comparison of flavonoid production in these three *in vitro*-cultured *H. perforatum* cultivars.

## Materials and Methods

### Origin of *in vitro* cultures

The *in vitro* shoot cultures of the ‘Elixir,’ ‘Helos,’ and ‘Topas’ cultivars of *H. perforatum* L. were established in 2007 at the Institute of Pharmaceutical Biology, Technische Universität Braunschweig (Germany). Details on the established shoot cultures were reported previously (Muszyńska et al. 2014).

### Initial *in vitro* cultures

The initial cultures were maintained on solid (Phyto agar, Duchefa Biochemie, Haarlem, The Netherlands) MS medium supplemented with 0.5 mg L^−1^ each of NAA and BAP (pH 5.7 adjusted with 1 M NaOH before autoclaving). Medium was sterilized for 20 min, at 121 °C and 0.1 MPa (SMS, Warszawa, Poland). The cultures were grown in Erlenmeyer flasks (250 mL) under constant artificial light with a light intensity 16 μmol m^−2^ s^−1^ (daylight, LF-40 W lamp, POLAMP, Giżycko, Poland) at 25 ± 2°C and were subcultured every 6 wk.

### Maintenance of agitated shoot cultures

Agitated shoot cultures of the three *H. perforatum* cultivars were maintained on four different variants of each LS medium and MS medium, which contained 0.1, 1.0, 2.0, or 3.0 mg L^−1^ each of NAA and BAP. Each Erlenmeyer flask (500 mL) contained 150 mL of medium. The agitated cultures were initiated with an inoculum of 1 g per flask of fresh biomass with one to three shoot clusters. The cultures (three replicate flasks) were maintained under the same conditions as the initial cultures, with agitation accomplished using a rotary shaker (Altel, Łódź, Poland) operating at 140 rpm with an amplitude of 35 mm for 3 wk., as previously described by Kwiecień et al. (2015). The biomass increment was calculated by dividing the sample dry weight by the dry weight of inoculum (dried 1 g of fresh biomass).

### Extraction and quantitative HPLC analysis

The biomass was collected at the end of the growth cycles (three replicate flasks), dried in fresh air at 25 ± 2°C (40–50% humidity), ground, and immediately extracted with 50 mL of methanol for 3 h at 78°C. The methanolic extracts were analyzed using an HPLC system (Elite LaChrome, L-2000 series, Hitachi, Tokyo, Japan) equipped with a Purospher RP-18e analytical column (4 × 250 mm, 5 μm, Merck, Darmstadt, Germany) at 25°C. The mobile phase consisted of methanol (A) and 0.5% (*v/v*) acetic acid (B). The flow rate was set on 1.0 mL min^−1^ with an injection volume 10 μL (modified after Ellnain-Wojtaszek and Zgórka 1999). The following gradient elution scheme was used: (A/B ratio) 20:80%, *t* = 0–20 min; 30–70%, *t* = 35 min; 60–40%, *t* = 60 min; 100–0%, *t* = 70–75 min; 20–80%, *t* = 80–90 min. Compounds were estimated using a diode-array detector (DAD) (Hitachi), at 254 nm (recording range of 200–400 nm). Quantification analyses were based on a comparison with reference substances (21 flavonoids and hypericin): apigenin, chrysin, cynaroside, quercetin, quercitrin, luteolin, mirycetin, rutoside, trifolin, vitexin, and hypericin (Sigma-Aldrich®, St Louis, MO), diosmetin (Roth, Karlsruhe, Germany), kaempferol (ChromaDex®, Irvine, CA), apigetrin, hyperoside, isorhamnetin, isorhamnetin 3-rhamnoside, kaempferol 3-rhamnoside, kaempferol 7-ramnoside, quercimetrinin, populnin, and rhamnetin (isolated in the Department of Pharmacognosy, Medical University of Gdańsk, Poland).

### Statistical analysis

The results were expressed as means ± standard deviation of three independent experiments. The analysis of variance (two-way ANOVA) was conducted for each metabolite content against cultivars of *H. perforatum* and culture medium variants. The model used in the analysis included the main effects and the interaction between them. After ANOVA analysis, the Duncan post hoc test was performed. The significance level was *p* < 0.05. Statistical analysis was performed using STATISTICA™ (TIBCO® Software Co., Palo Alto, CA) software.

## Results

### Variants of LS medium—behavior of biomass and accumulation of flavonoids

**Figure 1.**
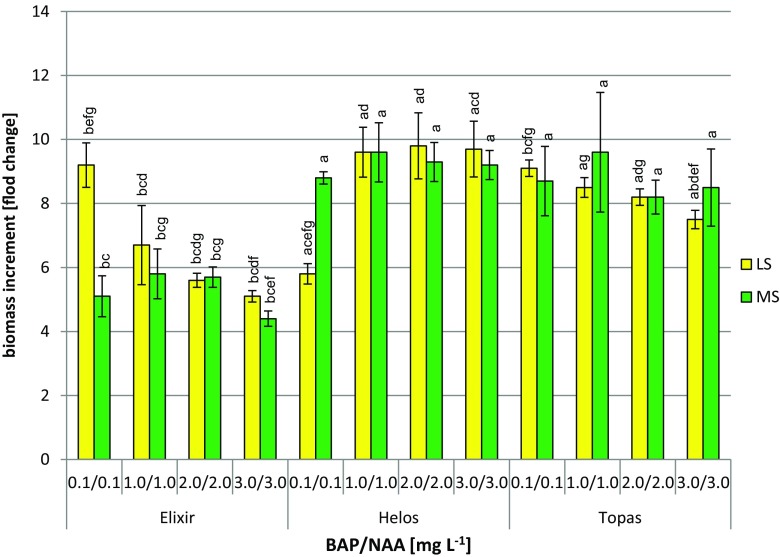
Dry biomass increments (fold change) of *Hypericum perforatum* cultivars cultured *in vitro* on LS (Linsmaier and Skoog 1965) and MS (Murashige and Skoog 1962) medium variants with different concentrations of 6-benzylaminopurine (BAP) and α-naphthaleneacetic acid (NAA) (3 series). The two-way analysis of variance. Statistically significant differences *p* < 0.05. For the same medium variant: (*a*) *vs.* Elixir; (*b*) *vs.* Helos; (*c*) *vs.* Topas. For the same cultivar: (*d*) *vs.* medium variant 0.1/0.1; (*e*) *vs.* medium variant 1.0/1.0; (*f*) *vs.* medium variant 2.0/2.0; (*g*) *vs.* medium variant 3.0/3.0

The biomass of agitated shoot cultures of *H. perforatum* cultivars ‘Elixir,’ ‘Helos,’ and ‘Topas,’ cultivated on the variants of LS medium tested, showed different increases in dry biomass during the 3-wk culture cycles. Dry biomass increments ranged from 5.1- to 9.2-fold for ‘Elixir,’ 5.8- to 9.8-fold for ‘Helos,’ and 7.5- to 9.1-fold for ‘Topas’ (Fig. 1). The highest increments (9.6- to 9.8-fold) were confirmed for ‘Helos’ grown on LS medium containing 1.0–3.0 mg L^−1^ BAP and NAA. Very high biomass increments for cultivars ‘Elixir’ and ‘Topas’ of 9.2- and 9.1-fold, respectively, were observed from LS medium supplemented with 0.1 mg L^−1^ BAP and 0.1 mg L^−1^ NAA.

**Figure 2.**
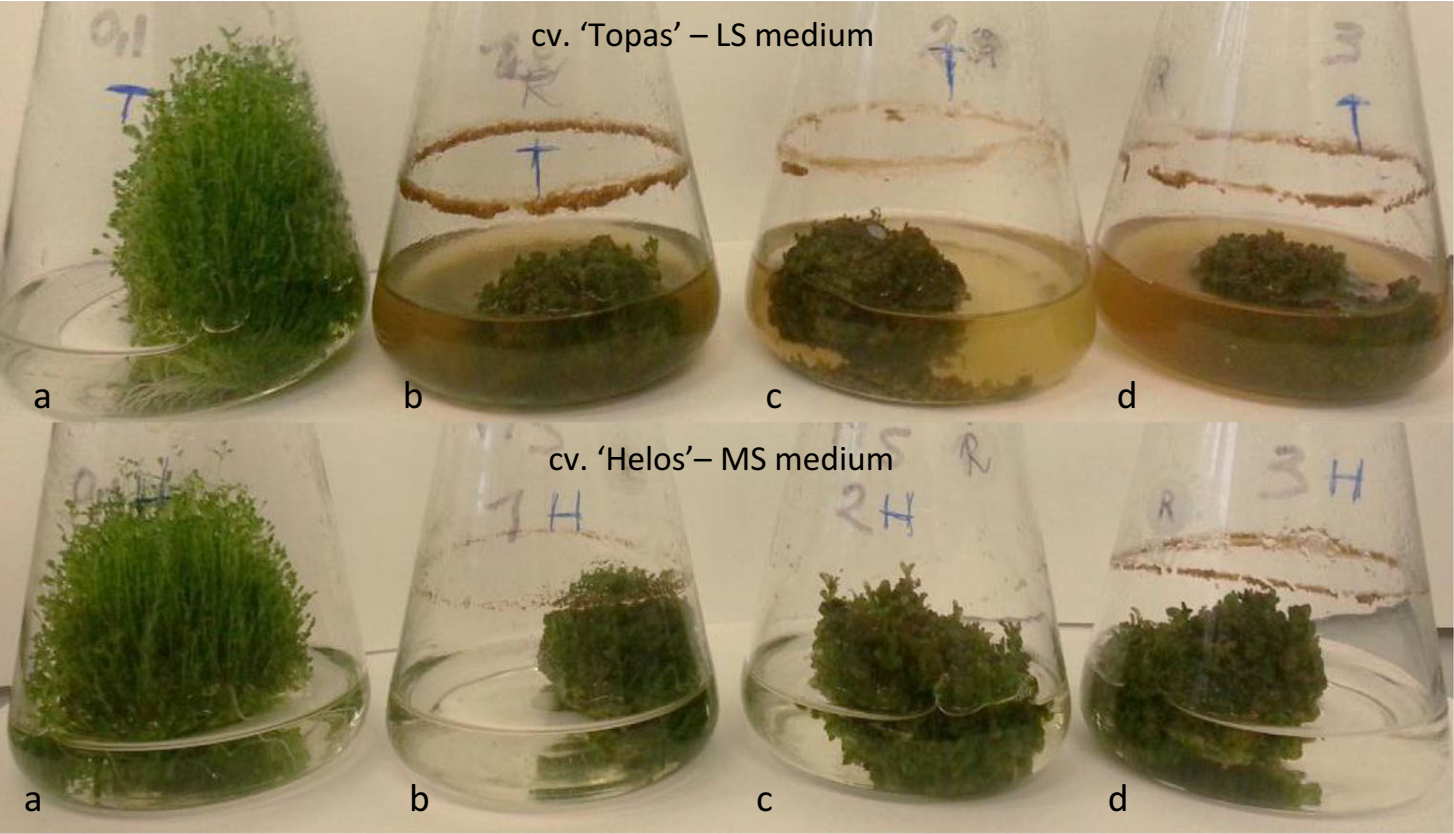
Experimental 3-wk-old agitated *in vitro* cultures of *Hypericum perforatum* cultivar ‘Topas’ on LS medium (Linsmaier and Skoog 1965) and cultivar ‘Helos’ on MS medium (Murashige and Skoog 1962). Medium variants containing: (*a*) 0.1/0.1 mg L^−1^ 6-benzylaminopurine (BAP) and α-naphthaleneacetic acid (NAA); (*b*) 1.0/1.0 mg L^−1^ BAP and NAA; (*c*) 2.0/2.0 mg L^−1^ BAP and NAA; (*d*) 3.0/3.0 mg L^−1^ BAP and NAA.

The shoots of the three cultivars cultured on the LS medium variants tested had different morphology (Fig. 2). Numerous green shoots were formed on the medium containing 0.1 mg L^−1^ BAP and 0.1 mg L^−1^ NAA, whereas on the other media supplemented with higher concentrations of PGRs, the shoots were shorter and formed a compact biomass of callus tissue at the base.

Following HPLC analyses of the extracts collected from the biomass of *H. perforatum* cultivars, all three were found to contain three aglycones (kaempferol, quercetin, and luteolin), and three glycosides of quercetin (hyperoside, quercitrin, and rutoside). None of the extracts analyzed contained any of the remaining 15 study compounds.

**Figure 3.**
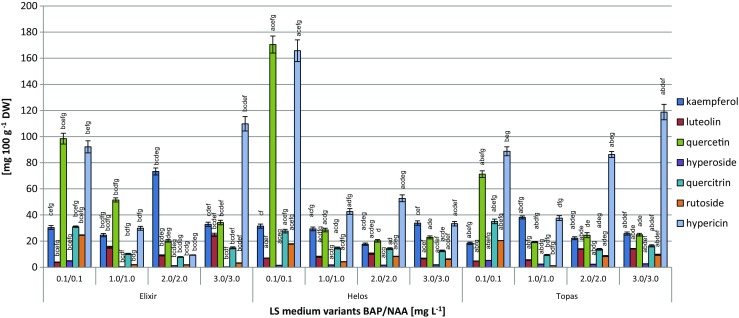
Content (mg 100 g^−1^ DW ± SD) of individual flavonoids and hypericin in biomass extracts from *Hypericum perforatum* cultivars cultured *in vitro* on LS medium (Linsmaier and Skoog 1965) variants with different concentrations of 6-benzylaminopurine (BAP) and α-naphthaleneacetic acid (NAA) (3 series). The two-way analysis of variance. Statistically significant differences *p* < 0.05. For the same medium variant: (*a*) *vs.* Elixir; (*b*) *vs.* Helos; (*c*) *vs.* Topas. For the same cultivar: (*d*) *vs.* medium variant 0.1/0.1; (*e*) *vs.* medium variant 1.0/1.0; (*f*) *vs.* medium variant 2.0/2.0; (*g*) *vs.* medium variant 3.0/3.0.

The levels of the individual metabolites and consequently, the total levels of flavonoids varied considerably, depending on the concentrations of PGRs in the LS medium tested (Table S1). The quercetin content varied within a very wide range, from 19.30 to 170.48 mg 100 g^−1^ DW. The confirmed amounts of kaempferol were less variable (18.28–73.32 mg 100 g^−1^ DW). The amounts of luteolin were decidedly smaller (4.71–24.75 mg 100 g^−1^ DW) (Fig. 3). The maximum levels of glycosides (quercitrin, rutoside, and hyperoside) did not exceed 35.5, 24.6, and 5.2 mg 100 g^−1^ DW, respectively. The amounts of glycosides in biomass extracts of the three cultivars on the tested LS media, also varied considerably (4.5-, 21.7-, and 17.3-fold, respectively) (Fig. 3).

The total amounts of the measured compounds increased on the tested LS media, 1.86-fold for ‘Elixir,’ 3.53-fold for ‘Helos,’ and 2.05-fold for ‘Topas.’ The maximum total amounts of flavonoids for ‘Elixir,’ ‘Helos,’ and ‘Topas’ of 193.05, 255.70, and 154.96 mg 100 g^−1^ DW, respectively, were observed for the LS medium with 0.1 mg L^−1^ BAP and 0.1 mg L^−1^ NAA. The highest total amount of flavonoids (over 100 mg 100 g^−1^ DW) was observed for cultivar ‘Elixir’ on all other LS medium variants tested (Table 1).

**Table 1.** Total content (mg 100 g^−1^ DW ± SD) of flavonoids in biomass extracts from *Hypericum perforatum* cultivars cultured *in vitro* on LS (Linsmaier and Skoog 1965) and MS (Murashige and Skoog 1962) medium variants with different concentrations of 6-benzylaminopurine (BAP) and α-naphthaleneacetic acid (NAA) (3 series)

*H. perforatum* cultivar	BAP/NAA (mg L^−1^)	Total flavonoids (mg 100 g^−1^ DW)	
		LS medium	MS medium
Elixir	0.1/0.1	193.05 ± 6.68bcefg	328.53 ± 9.43bcefg
	1.0/1.0	103.92 ± 4.14bcd	123.83 ± 5.00bcdfg
	2.0/2.0	113.37 ± 4.55bcd	160.32 ± 7.72bcdeg
	3.0/3.0	110.14 ± 5.51bcd	185.22 ± 8.36bcdef
Helos	0.1/0.1	255.7 ± 9.87acefg	146.82 ± 6.14acefg
	1.0/1.0	86.45 ± 4.21acdf	96.32 ± 4.33adf
	2.0/2.0	72.31 ± 3.28acdeg	106.19 ± 5.18acde
	3.0/3.0	83.83 ± 4.19adf	98.19 ± 4.62ad
Topas	0.1/0.1	154.97 ± 5.39abefg	166.58 ± 7.03abefg
	1.0/1.0	75.64 ± 2.49abdfg	98.62 ± 4.61adf
	2.0/2.0	85.23 ± 4.53abdeg	74.69 ± 3.69abdeg
	3.0/3.0	93.26 ± 3.81adef	93.16 ± 3.52adf

For the same medium variant: (a) *vs.* Elixir; (b) *vs.* Helos; (c) *vs.* Topas. For the same cultivar: (d) *vs.* medium variant 0.1/0.1; (e) *vs.* medium variant 1.0/1.0; (f) *vs.* medium variant 2.0/2.0; (g) *vs.* medium variant 3.0/3.0

The two-way analysis of variance. Statistically significant differences *p* < 0.05

The amounts of hypericin measured in the biomass of shoots of the three cultivars were markedly different and ranged from 9.31 to 165.79 mg 100 g^−1^ DW. The highest hypericin content was found in extracts from the shoots of cultivar ‘Helos’ growing on the LS medium supplemented with 0.1 mg L^−1^ each of BAP and NAA (Fig. 3).

### Variants of MS medium—behavior of biomass and accumulation of flavonoids

On the MS media tested, the biomass from agitated shoot cultures of the three cultivars *‘*Elixir,’ ‘Helos,’ ‘Topas,’ also showed varied increases during the 3-wk culture cycles, from 4.4- to 5.8-fold for ‘Elixir,’ 8.8- to 9.6-fold for ‘Helos,’ and 8.2- to 9.6-fold for ‘Topas’ (Fig. 1). The highest increases in biomass (over 8.2-fold) were observed with cultivars ‘Helos’ and ‘Topas’ grown on all of the MS media tested. The highest increases (9.6-fold) were shown by the shoots of these two cultivars, grown on the same MS medium containing 1.0 mg L^−1^ BAP and 1.0 mg L^−1^ NAA. The MS media tested were not favorable for biomass growth in cultivar ‘Elixir,’ which had a maximum increase of 5.8-fold.

As in the case of the LS medium variants, the morphology of the shoots from the three cultivars grown on MS media with various concentrations of PGRs was found to vary. On the MS medium containing 0.1 mg L^−1^ each of NAA and BAP, numerous green shoots were formed. With the increase in the concentrations of PGRs, the shoots became more compact and callus formed at their bases (Fig. 2).

The chromatographic analysis of extracts from the cultured biomass of the three *H. perforatum* cultivars confirmed the presence of the same compounds that were found on LS media - kaempferol, quercetin, luteolin, hyperoside, quercitrin, and rutoside.

**Figure 4.**
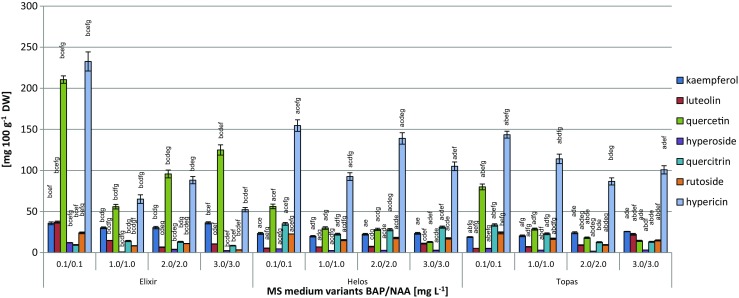
Content (mg 100 g^−1^ DW ± SD) of individual flavonoids and hypericin in biomass extracts from *Hypericum perforatum* cultivars cultured *in vitro* on MS medium (Murashige and Skoog 1962) variants with different concentrations of 6-benzylaminopurine (BAP) and α-naphthaleneacetic acid (NAA) (3 series). The two-way analysis of variance. Statistically significant differences *p* < 0.05. For the same medium variant: (*a*) *vs.* Elixir; (*b*) *vs.* Helos; (*c*) *vs.* Topas. For the same cultivar: (*d*) *vs.* medium variant 0.1/0.1; (*e*) *vs.* medium variant 1.0/1.0; (*f*) *vs.* medium variant 2.0/2.0; (g) *vs.* medium variant 3.0/3.0

On the MS media tested, as in the case of the LS media, there were also substantial differences in the amounts of individual compounds, depending on the concentrations of PGRs in the media (Table S2). Consequently, there were also large differences in the total amounts of flavonoids. Particularly marked differences in content were found in the case of quercetin (12.90–210.55 mg 100 g^−1^ DW). Considerably smaller quantitative differences were recorded for kaempferol (18.90–36.19 mg 100 g^−1^ DW) and luteolin (5.23–37.06 mg 100 g^−1^ DW) (Fig. 4). The amounts of quercitrin, rutoside, and hyperoside increased 4.2-, 7.5-, and 16.2-fold, respectively, and reached maximum values of 34.97, 23.99, and 12.00 mg 100 g^−1^ DW, respectively (Fig. 4).

The total amounts of the metabolites increased, depending on the MS medium, for each cultivar, which were 2.65-fold for ‘Elixir,’ 1.52-fold for ‘Helos,’ and 2.23-fold for ‘Topas.’ The highest total amounts of the estimated compounds in ‘Elixir,’ ‘Helos,’ and ‘Topas’ of 328.53, 146.82, and 166.57 mg 100 g^−1^ DW, respectively, were found for the MS medium containing 0.1 mg L^−1^ each of NAA and BAP (Table 1).

The analysis of the hypericin content in the biomass of the three cultivars grown on the MS media tested showed variable levels, ranging from 52.29 to 232.61 mg 100 g^−1^ DW. The maximum content was found in extracts from the biomass of cultivar ‘Elixir’ grown on MS medium supplemented with 0.1 mg L^−1^ BAP and 0.1 mg L^−1^ NAA (Fig. 4).

## Discussion

The observed increases in the dry biomass of all three cultivars in the agitated cultures were high. They were higher in comparison with increases in dry biomass of *Ruta graveolens* and *Ruta graveolens* ssp. *divaricata* agitated cultures growing on LS medium variants (Ekiert and Czygan 2005).

The tested concentrations of cytokinin (BAP) and auxin (NAA) in the media clearly affected the levels of the individual flavonoids and the total amounts. The influence of the composition and concentration of PGRs in the culture media on the accumulation of different groups of secondary metabolites was significant. It was documented for *in vitro* cultures of many medicinal plant species (Ramawat and Mathur 2007; Murthy et al. 2014). This was also demonstrated by previous studies which investigated the influence of different concentrations of BAP and NAA in LS and MS media variants, on the accumulation of groups of bioactive secondary metabolites including coumarins in callus cultures of *Pastinaca sativa*, *Ammi majus*, and *Anethum graveolens* (Ekiert and Gomółka 2000a, b; Szopa and Ekiert 2015b), schisandra lignans in shoot-differentiating callus cultures of *Schisandra chnensis* (Szopa and Ekiert 2011, 2013, 2015a), and also on the accumulation of phenolic acids in shoot cultures of *Ruta graveolens* and shoot-differentiating callus cultures of *R. graveolens* ssp. *divaricata* (Ekiert et al. 2009, 2014). The association of the levels of phenolic acids with concentrations of BAP and NAA in agitated shoot cultures of the three *H. perforatum* cultivars, which are the same biological materials for the present work (Kwiecień et al. 2015), was also demonstrated.

Concurrently, studies with *in vitro* cultures of *Aronia melanocarpa* (Szopa et al. 2013; Szopa and Ekiert 2014), and *in vitro* cultures of the three cultivars of *H. perforatum* (Kwiecień et al. 2015), showed the importance of the basic composition of the LS and MS media on the accumulation of phenolic acids. In the case of *in vitro* cultures of *A. melanocarpa* on MS media with the same concentrations of BAP and NAA, as in LS variants, 1.61- to 2.00-fold higher levels of phenolic acids were obtained (Szopa et al. 2013; Szopa and Ekiert 2014). In the study with *H. perforatum* cultivars, the results were different (Kwiecień et al. 2015). In only 4 out of the 12 extracts from the shoots grown on MS media, higher levels of phenolic acids were obtained (1.19- to 1.47-fold), compared to corresponding extracts from the shoots grown on identical LS medium variants (with the same concentrations of BAP and NAA as in MS medium variants).

In the present study on three *H. perforatum* cultivars, *in vitro* cultured higher total amounts of flavonoids in shoot extracts (1.07- to 1.71-fold) were found on nine MS media variants (out of 12) compared with the amounts from extracts of shoot grown on LS media with the same concentrations of BAP and NAA. These results documented that MS medium variants containing richer chemical compositions such as vitamins, compared to LS medium variants are generally more favorable for the biosynthesis and accumulation of flavonoids. Vitamins as cofactors of many enzymes can stimulate different enzymatic reactions in the cells of *in vitro* cultured shoots of the cultivars.

The present results demonstrated considerable levels of most individual flavonoids and consequently the total levels. This is undoubtedly associated with a high degree of differentiation of the *in vitro* cultured biomass. Many experiments documented this type of relationship (Charlwood et al. 1990). In previous studies high amounts of different groups of secondary metabolites were confirmed, such as coumarins in shoot cultures of *Ruta graveolens* (Ekiert et al. 2001; Ekiert and Czygan 2005), and in shoot-differentiating callus cultures of *R. graveolens* ssp. *divaricata* (Ekiert and Czygan 2005; Ekiert et al. 2005), schisandra lignans in shoot-differentiating callus cultures (Szopa and Ekiert 2011, 2013, 2015a), and also phenolic acids in shoot cultures of *Ruta graveolens* (Ekiert et al. 2009), and in shoot-differentiating callus cultures of *R. graveolens* ssp. *divaricata* (Ekiert et al. 2014). Also, in a previous study, the amounts of phenolic acids obtained from agitated shoot cultures of the *H. perforatum* cultivars ‘Elixir,’ ‘Helos,’ and ‘Topas’ were high (Kwiecień et al. 2015).

Various studies connected with the biosynthesis and accumulation of flavonoids documented that the richest source of the flavonoid fraction was shoot cultures. It was documented among others for shoot cultures of *H. perforatum* (Dias et al. 1999). The lower the degree of organogenesis (callus cultures with shoot buds, callus cultures, and cell suspensions), the lower the diversity, and overall flavonoid content (Dias et al. 1999; Pasqua et al. 2003; Bertoli et al. 2008).

Presented results clearly indicate that both the MS and the LS media with the lowest concentrations of PGRs (0.1 mg L^−1^) were the most favorable for the accumulation of flavonoids. The agitated shoot cultures of cultivar ‘Elixir’ grown on the MS medium variant containing 0.1 mg L^−1^ BAP and 0.1 mg L^−1^ NAA produced the richest source of flavonoids (328.5 mg 100 g^−1^ DW) (Table 1). A rich source of flavonoids was also obtained in cultures of cultivars ‘Helos’ and ‘Elixir’ maintained on the LS medium containing 0.1 mg L^−1^ each of NAA and BAP. This medium can be considered as a very good productive medium for ‘Elixir’ and ‘Helos.’ However, on both of these production media, the biomass growth increments were the lowest. On the other hand, on the same media variants, the level of differentiation was the highest (shoot cultures without callus). The total amounts of flavonoids produced on the abovementioned LS and MS media variants were the highest observed (146.82–328.53 mg 100 g^−1^ DW) for all cultivars (Table 1). Similar correlations were obtained earlier with experiments connected with accumulation of phenolic acids in the same three cultivars of *in vitro* cultured *H. perforatum*. The richest sources of flavonoids were shoots cultivated on LS and MS media variants with 0.1 mg L^−1^ each of BAP and NAA (Kwiecień et al. 2015).

The shoots of the cultivars studied showed a clear ability to accumulate high amounts of quercetin and moderate amounts of its glycosides, quercitrin, and rutoside. The amounts of hyperoside were low (Figs. 3 and 4). The maximum levels of quercetin, which is the main metabolite (approx. 210 mg 100 g^−1^ DW), were high enough to be interesting from practical point of view (Li et al. 2016). This compound has very valuable biological properties for therapy, anti-inflammatory, antioxidant, anticancer, and hepatoprotective properties. Other research teams that focused on the cultivar ‘Topas’ documented many fold lower levels of quercetin, but higher levels of rutoside and hyperosde, which are glycosides of this compound. These results were documented only for plantlets without roots regenerated from callus, and not for undifferentiated callus, and callus with vegetative bud cultures (Pasqua et al. 2003).

Cultivar ‘Elixir’ is characterized by the highest capacity to accumulate hypericin on MS medium containing 0.1 mg L^−1^ each of BAP and NAA. Current results demonstrated that levels of hypericin on all media tested for the three cultivars of *H perforatum* are much higher than in a previous study (Kwiecień et al. 2015). This could suggest that *in vitro* cultures adapt to the growing conditions and start to produce more complex metabolites compared to phenolic acids. Hypericin is a derivative of antraquinone with a dimeric structure. Accumulation of high levels of this compound is an important result because under *in vitro* conditions, a culture usually loses its biosynthetic capacity during successive grow cycles.

On the basis of earlier studies and the current results, it appears that cultivars ‘Elixir’ and ‘Helos’ possess significantly higher biosynthetic ability to produce phenolic acids and flavonoids, compared to the ‘Topas’ cultivar. To obtain the high levels of flavonoids, a two-step *in vitro* system is proposed. The first step should be the culture on growth promoting LS medium containing 0.1 mg L^−1^ BAP and 0.1 mg L^−1^ NAA for cultivar ‘Elixir’ and one of six LS and MS media variants, containing PGRs in a range from 1.0 to 3.0 mg L^−1^ for cultivar ‘Helos.’ The second step should be the culture on production medium MS for cultivar ‘Elixir’ and LS for cultivar ‘Helos,’ supplemented with 0.1 mg L^−1^ BAP and 0.1 mg L^−1^ NAA. The next proposal is an *in vitro* culture of cultivar ‘Topas’ cultured on LS or MS variant medium containing 0.1 mg L^−1^ BAP and 0.1 mg L^−1^ NAA. Both of these media compositions provide high growth increments and moderate biomass productivity for this cultivar to be a good potential source of bioactive flavonoids.

This is the first comparison of the production of flavonoids in three *in vitro* cultured *H. perforatum* cultivars. These results documented the diverse biosynthetic potential of *in vitro* cultured cultivars and their different ability to produce up to six different flavoniod substances, whose value as a potential pharmaceutical and/or cosmetological raw material varies. At the same time, this research demonstrates that the biomass grown *in vitro* can accumulate high levels of flavonoid compounds, which are valuable metabolites with proven antioxidant, anti-inflammatory, anti-aggregatory, anti-aging, and many other properties.

## Electronic supplementary material

ESM 1 (DOCX 44 kb)
