# Supplementary material for: The impact of media composition on production of flavonoids in agitated shoot cultures of the three Hypericum perforatum L. cultivars ‘Elixir,’ ‘Helos,’ and ‘Topas’
Source: In Vitro Cell Dev Biol Plant. 2018 Apr 9;54(3):332–40. doi: 10.1007/s11627-018-9900-7 (PMC5954008; doi:10.1007/s11627-018-9900-7)
Supplement: Supplementary file 1 — (DOCX 44 kb) [file 11627_2018_9900_MOESM1_ESM.docx]

**Supplementary material**

**Table 1.** Two-way ANOVA for compounds content in the dry biomass (Linsmaier & Skoog medium); SS - sum of squares; d.o.f. – degrees of freedom; MS – mean square; F – Fisher’s statistic; p – critical level of significance.

| Effect | Total flavonoids | | | | |
| --- | --- | --- | --- | --- | --- |
|  | \| SS \| \| --- \| | \| d.o.f. \| \| --- \| | \| MS \| \| --- \| | \| F \| \| --- \| | \| p \| \| --- \| |
| \| 1. PGRs (BAP and NAA) concentration \| \| --- \| | 81443,4 | 3 | 27147,8 | 1020,72 | 0,00000 |
| \| 2. cultivar \| \| --- \| | 5252,4 | 2 | 2626,2 | 98,74 | 0,00000 |
| \| 1 × 2 \| \| --- \| | 15240,1 | 6 | 2540,0 | 95,50 | 0,00000 |
| \| Error \| \| --- \| | 638,3 | 24 | 26,6 |  |  |

| Effect | Kaempferol | | | | |
| --- | --- | --- | --- | --- | --- |
|  | \| SS \| \| --- \| | \| d.o.f. \| \| --- \| | \| MS \| \| --- \| | \| F \| \| --- \| | \| p \| \| --- \| |
| \| 1. PGRs (BAP and NAA) concentration \| \| --- \| | 564,27 | 3 | 188,09 | 85,02 | 0,00000 |
| \| 2. cultivar \| \| --- \| | 1410,66 | 2 | 705,33 | 318,81 | 0,00000 |
| \| 1 × 2 \| \| --- \| | 5055,15 | 6 | 842,53 | 380,82 | 0,00000 |
| \| Error \| \| --- \| | 53,10 | 24 | 2,21 |  |  |

| Effect | Luteolin | | | | |
| --- | --- | --- | --- | --- | --- |
|  | \| SS \| \| --- \| | \| d.o.f. \| \| --- \| | \| MS \| \| --- \| | \| F \| \| --- \| | \| p \| \| --- \| |
| \| 1. PGRs (BAP and NAA) concentration \| \| --- \| | 462,358 | 3 | 154,119 | 622,20 | 0,00000 |
| \| 2. cultivar \| \| --- \| | 170,668 | 2 | 85,334 | 344,51 | 0,00000 |
| \| 1 × 2 \| \| --- \| | 535,274 | 6 | 89,212 | 360,16 | 0,00000 |
| \| Error \| \| --- \| | 5,945 | 24 | 0,248 |  |  |

| Effect | Quercetin | | | | |
| --- | --- | --- | --- | --- | --- |
|  | \| SS \| \| --- \| | \| d.o.f. \| \| --- \| | \| MS \| \| --- \| | \| F \| \| --- \| | \| p \| \| --- \| |
| \| 1. PGRs (BAP and NAA) concentration \| \| --- \| | 50509,64 | 3 | 16836,55 | 2480,86 | 0,00000 |
| \| 2. cultivar \| \| --- \| | 3988,63 | 2 | 1994,32 | 293,86 | 0,00000 |
| \| 1 × 2 \| \| --- \| | 13720,73 | 6 | 2286,79 | 336,96 | 0,00000 |
| \| Error \| \| --- \| | 162,88 | 24 | 6,79 |  |  |

| Effect | Hyperoside | | | | |
| --- | --- | --- | --- | --- | --- |
|  | \| SS \| \| --- \| | \| Stopnie swobody \| \| --- \| | \| MS \| \| --- \| | \| F \| \| --- \| | \| p \| \| --- \| |
| \| 1. PGRs (BAP and NAA) concentration \| \| --- \| | 34,5709 | 3 | 11,5236 | 2162,67 | 0,00000 |
| \| 2. cultivar \| \| --- \| | 16,3376 | 2 | 8,1688 | 1533,06 | 0,00000 |
| \| 1 × 2 \| \| --- \| | 25,5059 | 6 | 4,2510 | 797,79 | 0,00000 |
| \| Error \| \| --- \| | 0,1279 | 24 | 0,0053 |  |  |

| Effect | Quercitrin | | | | |
| --- | --- | --- | --- | --- | --- |
|  | \| SS \| \| --- \| | \| d.o.f. \| \| --- \| | \| MS \| \| --- \| | \| F \| \| --- \| | \| p \| \| --- \| |
| \| 1. PGRs (BAP and NAA) concentration \| \| --- \| | 2378,06 | 3 | 792,69 | 1088,67 | 0,00000 |
| \| 2. cultivar \| \| --- \| | 42,69 | 2 | 21,35 | 29,32 | 0,00000 |
| \| 1 × 2 \| \| --- \| | 195,90 | 6 | 32,65 | 44,84 | 0,00000 |
| \| Error \| \| --- \| | 17,47 | 24 | 0,73 |  |  |

| Effect | Rutoside | | | | |
| --- | --- | --- | --- | --- | --- |
|  | \| SS \| \| --- \| | \| d.o.f. \| \| --- \| | \| MS \| \| --- \| | \| F \| \| --- \| | \| p \| \| --- \| |
| \| 1. PGRs (BAP and NAA) concentration \| \| --- \| | 1804,807 | 3 | 601,602 | 10072,05 | 0,00000 |
| 2. cultivar | 25,152 | 2 | 12,576 | 210,55 | 0,00000 |
| \| 1 × 2 \| \| --- \| | 206,811 | 6 | 34,469 | 577,07 | 0,00000 |
| \| Error \| \| --- \| | 1,434 | 24 | 0,060 |  |  |

| Effect | Hypericin | | | | |
| --- | --- | --- | --- | --- | --- |
|  | \| SS \| \| --- \| | \| d.o.f. \| \| --- \| | \| MS \| \| --- \| | \| F \| \| --- \| | \| p \| \| --- \| |
| \| 1. PGRs (BAP and NAA) concentration \| \| --- \| | 35088,9 | 3 | 11696,3 | 858,61 | 0,00000 |
| \| 2. cultivar \| \| --- \| | 3068,1 | 2 | 1534,1 | 112,61 | 0,00000 |
| \| 1 × 2 \| \| --- \| | 30744,8 | 6 | 5124,1 | 376,16 | 0,00000 |
| \| Error \| \| --- \| | 326,9 | 24 | 13,6 |  |  |

**Table 2.** Two-way ANOVA for compounds content in the dry biomass (Murashige & Skoog medium); SS- sum of squares; d.o.f. – degrees of freedom; MS – mean square; F – Fisher’s statistic; p – critical level of significance.

| Effect | Total flavonoids | | | | |
| --- | --- | --- | --- | --- | --- |
|  | \| SS \| \| --- \| | \| d.o.f. \| \| --- \| | \| MS \| \| --- \| | \| F \| \| --- \| | \| p \| \| --- \| |
| \| 1. PGRs (BAP and NAA) concentration \| \| --- \| | 67678,7 | 3 | 22559,6 | 602,71 | 0,00000 |
| \| 2. cultivar \| \| --- \| | 64170,0 | 2 | 32085,0 | 857,19 | 0,00000 |
| \| 1 × 2 \| \| --- \| | 24211,5 | 6 | 4035,2 | 107,81 | 0,00000 |
| \| Error \| \| --- \| | 898,3 | 24 | 37,4 |  |  |

| Effect | Kaempferol | | | | |
| --- | --- | --- | --- | --- | --- |
|  | \| SS \| \| --- \| | \| d.o.f. \| \| --- \| | \| MS \| \| --- \| | \| F \| \| --- \| | \| p \| \| --- \| |
| \| 1. PGRs (BAP and NAA) concentration \| \| --- \| | 116,48 | 3 | 38,83 | 32,21 | 0,00000 |
| \| 2. cultivar \| \| --- \| | 932,63 | 2 | 466,32 | 386,83 | 0,00000 |
| \| 1 × 2 \| \| --- \| | 93,74 | 6 | 15,62 | 12,96 | 0,00000 |
| \| Error \| \| --- \| | 28,93 | 24 | 1,21 |  |  |

| Effect | Luteolin | | | | |
| --- | --- | --- | --- | --- | --- |
|  | \| SS \| \| --- \| | \| d.o.f. \| \| --- \| | \| MS \| \| --- \| | \| F \| \| --- \| | \| p \| \| --- \| |
| \| 1. PGRs (BAP and NAA) concentration \| \| --- \| | 421,878 | 3 | 140,626 | 427,19 | 0,00000 |
| \| 2. cultivar \| \| --- \| | 571,095 | 2 | 285,548 | 867,43 | 0,00000 |
| \| 1 × 2 \| \| --- \| | 1863,442 | 6 | 310,574 | 943,45 | 0,00000 |
| \| Error \| \| --- \| | 7,901 | 24 | 0,329 |  |  |

| Effect | Quercetin | | | | |
| --- | --- | --- | --- | --- | --- |
|  | \| SS \| \| --- \| | \| d.o.f. \| \| --- \| | \| MS \| \| --- \| | \| F \| \| --- \| | \| p \| \| --- \| |
| \| 1. PGRs (BAP and NAA) concentration \| \| --- \| | 34086,7 | 3 | 11362,2 | 1158,24 | 0,00000 |
| \| 2. cultivar \| \| --- \| | 62167,0 | 2 | 31083,5 | 3168,58 | 0,00000 |
| \| 1 × 2 \| \| --- \| | 16016,1 | 6 | 2669,3 | 272,11 | 0,00000 |
| \| Error \| \| --- \| | 235,4 | 24 | 9,8 |  |  |

| Effect | Hyperoside | | | | |
| --- | --- | --- | --- | --- | --- |
|  | \| SS \| \| --- \| | \| d.o.f. \| \| --- \| | \| MS \| \| --- \| | \| F \| \| --- \| | \| p \| \| --- \| |
| \| 1. PGRs (BAP and NAA) concentration \| \| --- \| | 151,8397 | 3 | 50,6132 | 2145,49 | 0,00000 |
| \| 2. cultivar \| \| --- \| | 20,1863 | 2 | 10,0932 | 427,85 | 0,00000 |
| \| 1 × 2 \| \| --- \| | 102,1241 | 6 | 17,0207 | 721,50 | 0,00000 |
| \| Error \| \| --- \| | 0,5662 | 24 | 0,0236 |  |  |

| Effect | Quercitrin | | | | |
| --- | --- | --- | --- | --- | --- |
|  | \| SS \| \| --- \| | \| d.o.f. \| \| --- \| | \| MS \| \| --- \| | \| F \| \| --- \| | \| p \| \| --- \| |
| \| 1. PGRs (BAP and NAA) concentration \| \| --- \| | 423,69 | 3 | 141,23 | 133,86 | 0,00000 |
| \| 2. cultivar \| \| --- \| | 1927,72 | 2 | 963,86 | 913,54 | 0,00000 |
| \| 1 × 2 \| \| --- \| | 789,10 | 6 | 131,52 | 124,65 | 0,00000 |
| \| Error \| \| --- \| | 25,32 | 24 | 1,06 |  |  |

| Effect | Rutoside | | | | |
| --- | --- | --- | --- | --- | --- |
|  | \| SS \| \| --- \| | \| d.o.f. \| \| --- \| | \| MS \| \| --- \| | \| F \| \| --- \| | \| p \| \| --- \| |
| \| 1. PGRs (BAP and NAA) concentration \| \| --- \| | 823,080 | 3 | 274,360 | 534,66 | 0,00000 |
| \| 2. cultivar \| \| --- \| | 279,281 | 2 | 139,640 | 272,13 | 0,00000 |
| \| 1 × 2 \| \| --- \| | 308,499 | 6 | 51,416 | 100,20 | 0,00000 |
| \| Error \| \| --- \| | 12,316 | 24 | 0,513 |  |  |

| Effect | Hypericin | | | | |
| --- | --- | --- | --- | --- | --- |
|  | \| SS \| \| --- \| | \| d.o.f. \| \| --- \| | \| MS \| \| --- \| | \| F \| \| --- \| | \| p \| \| --- \| |
| \| 1. PGRs (BAP and NAA) concentration \| \| --- \| | 48472,9 | 3 | 16157,6 | 464,29 | 0,00000 |
| \| 2. cultivar \| \| --- \| | 1227,6 | 2 | 613,8 | 17,64 | 0,00002 |
| \| 1 × 2 \| \| --- \| | 26790,2 | 6 | 4465,0 | 128,30 | 0,00000 |
| \| Error \| \| --- \| | 835,2 | 24 | 34,8 |  |  |
